# Emergence of Carbapenemase-Producing *Enterobacteriaceae*, South-Central Ontario, Canada^1^

**DOI:** 10.3201/eid2409.180164

**Published:** 2018-09

**Authors:** Philipp P. Kohler, Roberto G. Melano, Samir N. Patel, Shumona Shafinaz, Amna Faheem, Brenda L. Coleman, Karen Green, Irene Armstrong, Huda Almohri, Sergio Borgia, Emily Borgundvaag, Jennie Johnstone, Kevin Katz, Freda Lam, Matthew P. Muller, Jeff Powis, Susan M. Poutanen, David Richardson, Anu Rebbapragada, Alicia Sarabia, Andrew Simor, Allison McGeer, for the Toronto Invasive Bacterial Diseases Network (TIBDN)

**Affiliations:** Sinai Health System, Toronto, Ontario, Canada (P.P. Kohler, S. Shafinaz, A. Faheem, B.L. Coleman, K. Green, E. Borgundvaag, S.M. Poutanen, A. McGeer);; Public Health Ontario Laboratories, Toronto (R.G. Melano, S.N. Patel);; University of Toronto, Toronto (R.G. Melano, S.N. Patel, B.L. Coleman, I. Armstrong, J. Johnstone, K. Katz, M.P. Muller, J. Powis, S.M. Poutanen, A. Sarabia, A. Simor, A. McGeer);; Toronto Public Health, Toronto (I. Armstrong); LifeLabs, Toronto (H. Almohri);; McMaster University, Hamilton, Ontario, Canada (S. Borgia);; William Osler Health System, Brampton, Ontario, Canada (S. Borgia, D. Richardson);; St. Joseph’s Health Centre, Toronto (J. Johnstone);; Public Health Ontario, Toronto (J. Johnstone, K. Katz, F. Lam);; North York General Hospital, Toronto (K. Katz);; St. Michael’s Hospital, Toronto (M.P. Muller); Toronto East Health Network, Toronto (J. Powis);; University Health Network, Toronto (S.M. Poutanen); Dynacare, Brampton (A. Rebbapragada);; Trillium Health Partners, Toronto and Mississauga, Ontario, Canada (A. Sarabia);; Sunnybrook Health Sciences Centre, Toronto (A. Simor)

**Keywords:** carbapenem-resistant Enterobacteriaceae, CPE, drug resistance, bacteria, bacterial infections, beta-lactam resistance, population surveillance, incidence, Ontario, Canada, antimicrobial resistance

## Abstract

We analyzed population-based surveillance data from the Toronto Invasive Bacterial Diseases Network to describe carbapenemase-producing *Enterobacteriaceae* (CPE) infections during 2007–2015 in south-central Ontario, Canada. We reviewed patients’ medical records and travel histories, analyzed microbiologic and clinical characteristics of CPE infections, and calculated incidence. Among 291 cases identified, New Delhi metallo-β-lactamase was the predominant carbapenemase (51%). The proportion of CPE-positive patients with prior admission to a hospital in Canada who had not received healthcare abroad or traveled to high-risk areas was 13% for patients with oxacillinase-48, 24% for patients with New Delhi metallo-β-lactamase, 55% for patients with *Klebsiella pneumoniae* carbapenemase, and 67% for patients with Verona integron-encoded metallo-β-lactamase. Incidence of CPE infection increased, reaching 0.33 cases/100,000 population in 2015. For a substantial proportion of patients, no healthcare abroad or high-risk travel could be established, suggesting CPE acquisition in Canada. Policy and practice changes are needed to mitigate nosocomial CPE transmission in hospitals in Canada.

The global emergence of carbapenemase-producing *Enterobacteriaceae* (CPE) poses a threat to the achievements of modern medicine. The Centers for Disease Control and Prevention and the World Health Organization have recently classified CPE as one of the most urgent antimicrobial-resistance threats (*1*,*2*). CPE rarely arise de novo; rather, colonization and infection occur as a result of transmission of organisms, plasmids, or transposons from person to person, with such transmission occurring predominantly in healthcare institutions. An understanding of the epidemiology of the emergence of CPE and the changing burden over time is critical to the implementation of control programs and the management of individual patients.

In Canada, CPE were first reported in 2008 and have until recently been limited to individual cases and small outbreaks (*3*–*8*). Laboratory surveillance suggests substantial geographic variability, with *Klebsiella pneumoniae* carbapenemase (KPC) predominating in Quebec, whereas New Delhi metallo-β-lactamase (NDM) is most frequent in British Columbia (*9*,*10*). Nationally, time trends for CPE are discrepant; data from Canada’s Nosocomial Infection Surveillance Program suggest stable CPE numbers in recent years, but data from voluntary laboratory reporting indicate a clear increase (*11*–*13*). To avoid the limitations of these surveillance systems and to better assess changes in disease burden and epidemiology in Ontario, we analyzed data from population-based surveillance for CPE.

## Methods

### Setting

Metropolitan Toronto (Toronto) and the Regional Municipality of Peel (Peel) are adjacent municipalities in south-central Ontario, Canada; the 2016 populations were 2.7 million for Toronto and 1.4 million for Peel (*14*,*15*). The Toronto Invasive Bacterial Diseases Network (TIBDN) is a collaborative network of microbiology laboratories, infection-control practitioners, and public health departments that performs population-based surveillance for infectious diseases in Toronto and Peel. TIBDN laboratories provide service to all hospitals and >87% of long-term care homes and physician offices serving area residents. Among TIBDN hospitals, 13% (3/23) perform admission screening for CPE colonization for all previously hospitalized patients, and an additional 65% (15/23) screen only if patients have been hospitalized outside of Canada (A. Jamal, Sinai Health System, unpub. data, 2018).

### Data Sources

In Ontario, cases and clusters of CPE were first reported in 2008 (*16*). In 2011, guidelines for laboratory identification of CPE were published, and voluntary reporting to Public Health Ontario was initiated. In July 2014, TIBDN started active, population-based surveillance for laboratory-confirmed episodes of colonization or infection attributable to CPE. To identify CPE colonized or infected patients before July 2014, TIBDN laboratories and infection prevention and control programs accessed data from voluntary surveillance, searched microbiology laboratory databases for meropenem-nonsusceptible *Enterobacteriaceae*, reviewed hospital infection control department line lists and databases, and analyzed data from annual antimicrobial resistance reports from the Ontario Institute for Quality Management in Healthcare (IQMH). In addition, all isolates submitted for confirmatory testing to the Public Health Ontario Laboratory (PHOL), Canada’s National Microbiology Laboratory (NML), and the Canadian Nosocomial Infection Surveillance Program were identified. During active surveillance, each newly identified case in TIBDN laboratories was reported to the central study office, with annual audits of participating and reference laboratories conducted to ensure complete case identification (*17*). Patient information was reviewed for each isolate to ensure that patients were counted only once. 

### Laboratory Identification of CPE

All 18 TIBDN laboratories are accredited by IQMH and follow IQMH recommendations for CPE identification, which include screening of all clinical isolates with an ertapenem MIC >1 mg/L or a meropenem disc diffusion diameter <25 mm. Before 2010, laboratories (including PHOL) used the modified Hodge test for screening; during 2010–2015, laboratories either sent all such isolates to PHOL for confirmation (n = 7) or screened with the modified Hodge test (n = 1), the ROSCO KPC + MBL confirm ID KIT (Rosco Diagnostica, Taastrup, Denmark) (n = 9), or by direct in-house PCR (n = 1) (*18*). All isolates with a positive screen in all years were tested by PCR for the presence of *bla*_KPC_, *bla*_OXA-48_–like, *bla*_VIM_, *bla*_NDM_, *bla*_IMP_, and *bla*_SME_ genes at either PHOL (16 laboratories) (*19*) or NML (2 laboratories) (*20*).

For laboratory specimens yielding CPE, we recorded date of collection, body site, bacterial species, carbapenemase gene (or genes), reason for collection (i.e., screening versus clinical), and results of susceptibility testing. We used the first isolate from each patient to describe the distribution of bacterial species and carbapenemases. We reviewed charts associated with all isolates to identify CPE infections.

### Data Collection and Definitions

We collected data by performing chart review for all patients. We approached patients first identified on or after January 1, 2013, to obtain consent, and we collected additional data by conducting interviews with patients or with next of kin if the patient was deceased or otherwise not able to provide information. We used a standard case report form to extract data from hospital or office charts from the admission or outpatient visit during which CPE was first identified and for any TIBDN hospital admissions in the prior year. We recorded demographic information, postal code of residence, co-occuring conditions (including Charlson index score) (*21*), antimicrobial drug use, proton-pump and immunosuppressive therapies, surgeries, intensive care unit admissions, and medical interventions.

We collected dates, hospital names, country, and reason for consultation for healthcare contacts within and outside of Canada in the year before the culture that identified each patient as being CPE colonized or infected. We obtained travel history within 1 year before CPE detection from patient interviews conducted by study staff or infection control practitioners. We defined high-risk travel as travel to the Indian subcontinent (India, Sri Lanka, Bangladesh, Pakistan, and Afghanistan) (*22*,*23*).

For bacteremia, a positive blood culture result sufficed for the diagnosis of infection. For all other culture sites, we defined infection as the presence of a positive clinical culture, a chart-documented physician diagnosis, and the initiation of targeted antimicrobial therapy. We calculated the 30-day mortality rate starting from the date the relevant clinical culture was obtained.

### Statistical Analysis

We used SAS University Edition (SAS Institute, Cary, NC) for statistical analyses. We reported categorical variables as frequencies and proportions and continuous variables as median with interquartile range. We used χ^2^ or Fisher exact test, as appropriate, for comparison of dichotomous variables. For continuous variables, we used the Mann-Whitney U or Kruskal-Wallis test. We used the Benjamini and Hochberg procedure, with a false discovery rate of <0.05, to correct for multiple comparisons (*24*). We calculated incidence of CPE infection and bacteremia by using the first CPE infection or bacteremia from each resident of Toronto and Peel, on the basis of population estimates from Statistics Canada (*25*). We performed Poisson regression to assess time trends for all CPE infections, bloodstream infections, and sterile sites or urine isolates (*26*). We considered p values <0.05 statistically significant.

## Results

### Incidence and Outcome of CPE Infections

We identified 291 residents of Toronto or Peel who were colonized or infected with CPE during October 2007–December 2015. Charts were not available for 21 patients, and 12 patients declined consent. Among the remaining 258 patients, median age was 70 years (range 3 months–95 years), and 65% were male. Overall, 149 (58%) patients had >1 clinical isolate, and 92 (36%) had an infection caused by CPE. Urinary tract infections (n = 75 [82%]) were most common, followed by pneumonia and primary bacteremia (n = 13 [14%] each) (Table 1). Thirty-day mortality was 16% (15/92) for all infected patients and 31% (9/29) for patients with primary (5/13) or secondary (4/16) bacteremia.

**Table 1:** Isolate source and infection type among patients colonized or infected with carbapenemase-producing *Enterobacteriaceae*, Metropolitan Toronto and the Regional Municipality of Peel, south-central Ontario, Canada, 2007–2015*

Characteristic	No. (%)				
	All patients, n = 258	*Escherichia coli*, n = 86	*Klebsiella pneumonia*e, n = 122	*Enterobacter* spp., n = 30	Other,† n = 20
Isolate source‡					
Only screening	115 (45)	58 (67)	47 (39)	6 (20)	4 (20)
>1 clinical	149 (58)	30 (35)	79 (65)	24 (80)	16 (80)
Positive specimen types at first identification§					
Rectal or colostomy	138 (54)	61 (71)	64 (53)	9 (30)	4 (20)
Urine	89 (35)	19 (22)	52 (43)	11 (37)	7 (35)
Blood	21 (8)	4 (5)	9 (7)	3 (10)	5 (25)
Wound	15 (6)	2 (2)	10 (8)	1 (3.3)	2 (10)
Sputum or broncoalveolar lavage	12 (5)	1 (1)	6 (5)	3 (10)	2 (10)
Other	17 (7)	4 (5)	5 (4)	6 (20)	2 (10)
Infection‡					
Any	92 (36)	21¶ (24)	46 (38)	13 (43)	12 (60)
Urinary tract	75 (29)	19 (22)	46 (38)	6 (20)	4 (20)
Pneumonia	13 (5)	4 (5)	3 (3)	4 (13)	2 (10)
Other#	13 (5)	3 (4)	5 (4)	3 (10)	2 (15)
Primary bacteremia**	13 (5)	2 (2)	4 (3)	2 (7)	1 (5)

*Among first patient isolates; sums of specimen types exceed the number of patients because >1 specimen type may have yielded carbapenemase-producing *Enterobacteriaceae*. †*Citrobacter* spp. (n = 7), *Morganella morganii* (n = 4), *Serratia marcescens* (n = 4), *Klebsiella oxytoca* (n = 3), *Providencia rettgeri* (n = 1), *Proteus mirabilis* (n = 1). ‡Including all follow-up isolates available and all infections during the patients' hospitalization. §Including isolates from all specimens obtained within 2 days of the first positive specimen. ¶One patient originally colonized with a carbapenemase-producing *E. coli* subsequently experienced an infection with a carbapenemase-producing *Enterobacter cloacae.* #Includes 7 skin or soft tissue infections, 5 bone or joint infections, and 1 abdominal infection. **Patients with secondary bacteremia were classified according to their primary source of infection (urinary tract [n = 12] and pneumonia [n = 6]). Two bacteremic patients had both urinary tract infection and pneumonia diagnosed.

**Figure 1:**
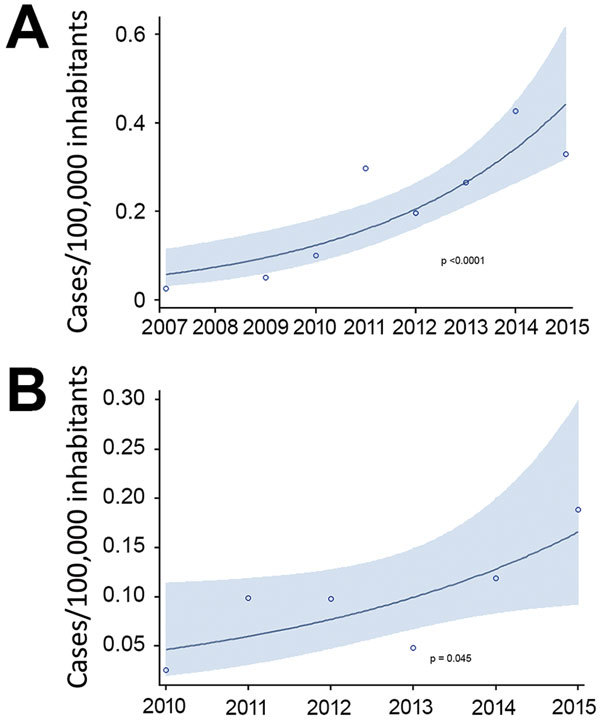
Incidence of all carbapenemase-producing enterobacterial infections per 100,000 inhabitants, 2007–2015 (A), and bloodstream infections per 100,000 inhabitants, 2010–2015 (B), calculated by using a Poisson regression model, Metropolitan Toronto and the Regional Municipality of Peel, south-central Ontario, Canada, 2007–2015. Shading indicates 95% CI.

The incidence of all CPE infections increased from 0 before 2007 to 0.33 cases/100,000 population in 2015 (p<0.0001); incidence of CPE bloodstream infections (primary and secondary) increased from 0 before 2007 to 0.19 cases/100,000 population in 2015 (p = 0.045) (Figure 1). For patients with >1 sterile site (i.e., blood, pleural or peritoneal space, or bone) or urine isolate, the incidence in 2015 was 0.52 cases/100,000 population.

### Patient Factors Associated with CPE Acquisition

In the year before CPE identification, 67% of patients had received antimicrobial drugs, 35% had undergone >1 surgical procedure, and 30% had had an intensive care unit admission. Overall, 71% (183/258) of CPE infections were categorized as hospital acquired (*27*). Risk profiles differed somewhat between patients with different carbapenemases (Table 2).

**Table 2:** Characteristics of patients with carbapenemase-producing *Enterobacteriaceae* infections, by type of carbapenemase, Metropolitan Toronto and the Regional Municipality of Peel, south-central Ontario, Canada, 2007–2015*

Patient characteristics and risk profile	All patients, n = 258†	NDM, n = 145	KPC, n = 64	OXA-48, n = 32	VIM, n = 12	p value‡
Sex						
M	168 (65)	94 (65)	37 (58)	25 (78)	8 (67)	0.32
F	90 (35)	51 (35)	27 (42)	7 (22)	4 (33)	
Age, y, median (IQR‡)	70 (57–79)	70 (59–79)	70 (50–79)	70 (52–77)	77 (65–88)	0.37
Charlson index score >2§	88 (34)	47 (32)	25 (39)	7 (22)	7 (58)	0.15
Inpatient at time of diagnosis	233 (90)	129 (89)	58 (91)	29 (91)	12 (100)	0.85
Days from admission to diagnosis, median (IQR)¶	2.5 (0–21)	0 (0–11)	14 (0–41)	0 (0–11)	19 (5–67)	**0.03**
CPE acquisition according to SHEA definitions#						
Hospital acquired, hospital onset	113 (44)	55 (38)	35 (55)	12 (38)	8 (67)	0.10
Hospital acquired, community onset	70 (27)	41 (28)	21 (33)	4 (13)	3 (25)	0.24
Undetermined	58 (23)	40 (28)	7 (11)	11 (34)	0	**0.024**
Community acquired	17 (7)	9 (6)	1 (2)	5 (16)	1 (8)	0.12
Residing in long-term care facility	9 (4)	2 (2)	4 (7)	0	3 (25)	**0.018**
Healthcare abroad or high-risk travel**	142/238 (60)	98/135 (73)	22/59 (37)	19/27 (70)	3/12 (25)	**0.0012**
Exposures and medical interventions††						
Intensive care stay	78 (30)	39 (27)	26 (41)	6 (19)	5 (42)	0.13
Mechanical ventilation	52 (20)	24 (17)	20 (31)	3 (9)	3 (25)	0.11
Previous surgery	91 (35)	29 (20)	41 (64)	13 (41)	4 (33)	**0.0012**
Central venous catheter	86 (33)	41 (28)	32 (50)	9 (28)	2 (17)	**0.03**
Antibiotic exposure, any	173 (67)	92 (64)	51 (80)	16 (50)	10 (83)	**0.03**
3rd- and 4th-generation cephalosporins	74 (29)	40 (28)	18 (28)	7 (22)	7 (58)	0.16
Carbapenems	33 (13)	14 (10)	12 (19)	3 (9)	3 (25)	0.17
Quinolones	81 (31)	41 (28)	26 (41)	6 (19)	7 (58)	0.05

*Values are no. (%) except as indicated. All characteristics and risk profile descriptors apply to the 1-year period preceding CPE detection. CPE, carbapenemase-producing *Enterobacteriaceae*; IQR, interquartile range; KPC, *Klebsiella pneumoniae* carbapenemase; NDM, New Delhi metallo-β-lactamase; OXA-48, oxacillinase 48; SHEA, Society for Healthcare Epidemiology of America; VIM, Verona integron-encoded metallo-β-lactamase. †Three patients with *Serratia marcescens* enzyme and 2 with non–metallo-carbapenemase are not listed separately. ‡p values corrected for multiple testing with the Hochberg and Benjamini procedure. Bold type indicates statistical significance (p<0.05). §No significant differences observed for any comorbid conditions. ¶Only patients included where first isolate is a clinical sample (n = 126). #Defined as hospital acquired if hospital admission occurred within 90 days before CPE detection. **High-risk countries and the Indian subcontinent. Denominators indicate no. patients with travel information available. ††Not listed because of nonsignificance: bronchoscopy, cystoscopy, dialysis, Foley catheter, urostomy, colostomy, tracheostomy, blood transfusion, proton-pump inhibitors, steroids, chemotherapy, immunosuppression, previously identified antibiotic-resistant pathogens (e.g., methicillin-resistant *Staphylococcus aureus* and extended-spectrum β-lactamase).

Travel history was available for 238 patients (92% of patients for whom clinical data were available); information was collected through patient interviews by study staff for 93 patients (39%) and from infection prevention and control staff for 145 patients (61%). A total of 142 patients (60%) had received healthcare abroad (n = 111) or reported travel to high-risk countries without a healthcare encounter (n = 31). Among these patients, 95/97 (97%) with NDM-producing isolates and 14/19 (74%) with oxacillinase 48 (OXA-48)–producing isolates reported travel to the Indian subcontinent with or without a healthcare encounter. In contrast, 15 (68%) of 22 patients with KPC-producing isolates had received healthcare in the United States or southern Europe, and 2 of 3 patients with Verona integron-encoded metallo-β-lactamase (VIM)–producing isolates had been admitted to hospitals in Croatia (n = 1) and Portugal (n = 1).

**Figure 2:**
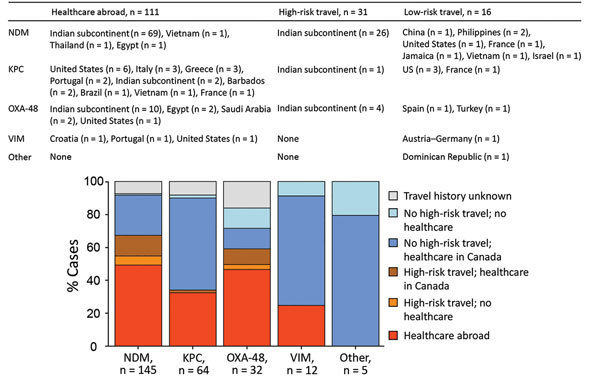
Healthcare visits abroad and travel history in patients with carbapenemase-producing *Enterobacteriaceae* infection in the 1 year before detection, stratified by type of carbapenemase, Metropolitan Toronto and the Regional Municipality of Peel, south-central Ontario, Canada, 2007–2015. Patients who traveled to any location other than the Indian subcontinent were classified as low-risk travel and indicated as no high-risk travel in the graph. n values indicate number of patients. KPC, *Klebsiella pneumoniae* carbapenemase; NDM, New Delhi metallo-β-lactamase; OXA-48, oxacillinase 48; VIM, Verona integron-encoded metallo-β-lactamase.

The proportion of CPE-positive patients with prior admission to a hospital in Canada who had not received healthcare abroad or traveled to high-risk areas was 13% for patients with OXA-48, 24% for patients with NDM, 55% for patients with KPC, and 67% for patients with VIM (p = 0.001). Of the 17 patients without healthcare encounters in Ontario or elsewhere (i.e., patients with presumptive community-acquired CPE), 9 (8 with NDM and 1 with OXA-48) reported high-risk travel in the year before CPE identification. Of an additional 8 patients (4 with OXA-48 and 1 each with NDM, KPC, *Serratia marcescens* enzyme, and VIM), 4 had detailed interviews conducted by study staff and reported neither healthcare exposure nor high-risk travel (Figure 2).

### Microbiology

**Figure 3:**
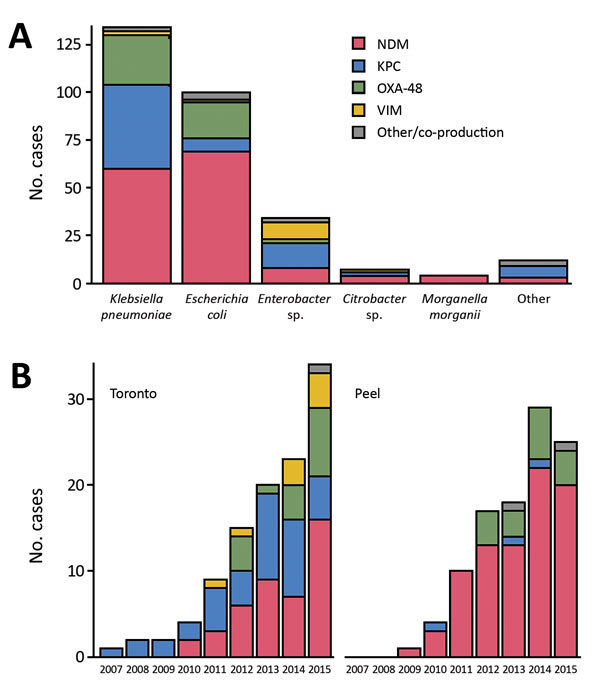
Distribution of carbapenemases in 291 first isolates of carbapenemase-producing *Enterobacteriaceae*, by enterobacterial species (A) and region (B), Metropolitan Toronto and the Regional Municipality of Peel, south-central Ontario, Canada, 2007–2015. Other enterobacterial species were *Serratia marcescens* (n = 4), *Klebsiella oxytoca* (n = 3), *Providencia rettgeri* (n = 1), and *Proteus mirabilis* (n =1). Other carbapenemases or co-productions were NDM–OXA-48 (n = 2) and *S. marcescens* enzyme (n = 1). KPC, *Klebsiella pneumoniae* carbapenemase; NDM, New Delhi metallo-β-lactamase; OXA-48, oxacillinase 48; VIM, Verona integron-encoded metallo-β-lactamase.

Overall, NDM was the most common carbapenemase (148/291 isolates [51%]), followed by KPC (72/291 isolates [25%]). NDM was most commonly found in *Escherichia coli* (69/148 isolates [47%]) and *K. pneumoniae* (60/148 isolates [41%]), whereas KPC was found predominantly in *K. pneumoniae* (44/72 isolates [61%]). The type of carbapenemases varied considerably over time and between Toronto and Peel (Figure 3).

Fourteen percent (12/86) of tested isolates were susceptible to nitrofurantoin, 14% (18/131) to ciprofloxacin, 25% (36/142) to trimethoprim/sulfamethoxazole, 30% (43/142) to gentamicin, 52% (15/29) to tigecycline, and 88% (15/17) to colistin. Isolates containing NDM genes were less susceptible to all antimicrobial drugs than isolates with other carbapenemase genes (Technical Appendix).

## Discussion

Since the first detection of CPE in Ontario in 2007, the incidence of CPE infections has been increasing steadily. Most patients with CPE had a recent history of healthcare abroad or travel to high-risk countries; NDM and OXA-48 producers were associated with travel in the Indian subcontinent and KPC producers with healthcare encounters in the United States and Mediterranean countries (*22*,*23*). However, a notable proportion of CPE patients had received healthcare in Canada but had no history of healthcare or travel abroad, suggesting that CPE transmission is occurring in Canada. The small number of patients without a history of healthcare abroad or high-risk travel might represent community acquisition in Canada but might also have resulted from travel or healthcare encounters that occurred >1 year before CPE detection.

Measuring population-based incidence is key to understanding the burden of disease and prioritizing public health interventions; however, population-based surveillance for CPE is complex and has rarely been performed. A non–population-based US study using 2012–2013 data estimated that the population incidence of CPE from urine or sterile sites combined was 1.4 cases/100,000 population (*26*). In our study, the incidence of urine or sterile site CPE isolates was 0.5 cases/100,000 population for 2015, which is ≈40% of the overall US CPE incidence and higher than the incidence in Oregon or New Mexico. Comparing this incidence in Canada with incidence elsewhere in the world is difficult because of the lack of published data; nevertheless, our data emphasize the steady increase and the geographic variability in CPE occurrence.

In immediately adjacent urban areas in south-central Ontario, substantial differences exist in the incidence and epidemiology of CPE infection. The higher incidence of NDM producers in Peel is probably associated with the fact that ≈28% of the local population is of South Asian descent compared with ≈12% in Toronto (*14*,*15*). Our finding that 51% of NDM carriers had healthcare encounters and an additional 21% reported travel to the Indian subcontinent supports the hypothesis that NDM is often introduced from these highly endemic countries. In contrast, patients with KPC and VIM producers more often do not have a history of high-risk travel or healthcare abroad, suggesting that CPE was acquired in hospitals in Canada. The facts that 1) KPC and VIM most commonly occurred in species associated with hospital-acquired infections (*K. pneumoniae* and *Enterobacter* spp.) whereas *E. coli*, the main cause of community-acquired enterobacterial infections, almost exclusively harbored NDM and OXA-48; 2) clinical isolates producing KPC or VIM were detected later in the course of hospitalization; and 3) most patients with KPC producers had had previous surgery or a central venous catheter, are consistent with other studies and with these isolates having been acquired during hospital admission (*28*,*29*). Similarly, in a Germany study, a higher proportion of patients with OXA-48 had traveled before CPE detection compared with patients with VIM, suggesting nosocomial acquisition of VIM producers (*30*). In a multicenter study conducted in 34 hospitals in Spain, VIM producers were also more likely to be hospital acquired than OXA-48 producers (*31*).

The fact that most CPE in our study population appear to be acquired in healthcare settings strongly suggests that intensification of control programs in this population is needed if we wish to protect patients from the impacts of CPE (*32*,*33*). Although the cost of control programs is a concern, the relatively low incidence of CPE in our population should be an incentive to implement such programs; control programs have been shown to be cost-effective in low-prevalence areas (*34*,*35*), and success in transmission control programs is more likely when they are implemented while prevalence of colonization is low (*33*). Our data are consistent with a recent assessment of CPE transmission in England; although we might perceive that large problems in India pose the greatest risk, the much larger number of our patients exposed to a smaller problem in Ontario likely poses the greater risk to our patient population (*36*).

Relative to isolates from other countries, CPE isolates in Toronto and Peel are more susceptible to commonly used antimicrobial drugs (*37*). Nonetheless, most isolates are resistant to all commonly used orally available antimicrobial drugs, and choices for parenteral therapy are limited. These concerns emphasize the need for the continued development of new antimicrobial drugs active against these resistant organisms.

Our study has several limitations. Although laboratory testing in Ontario is standardized, the modified Hodge test, the only screening test available before 2011, might have missed a small number of CPE during this period. However, PCR screening of all meropenem-nonsusceptible *Enterobacteriaceae* isolates from 4 TIBDN teaching hospitals during 2009–2011 at NML identified only a single additional CPE. Further, the increase in CPE infection incidence during 2010–2015 remains statistically significant. Because most TIBDN hospitals screen only patients who have accessed healthcare outside of Canada, our data on colonization will be biased toward the identification of CPE in these populations. This bias will underestimate the number of patients with colonization acquired in Canada. Similarly, our surveillance system detects only laboratory-confirmed infections, and infections for which cultures are not obtained will have been missed. This misclassification error might be lower for CPE than other organisms because resistance by CPE means that they might fail empiric therapy. We used a definition of high-risk countries for travel and healthcare currently used in Ontario hospitals (A. Jamal, Sinai Health System, unpub. data, 2018), but surveillance data are not available for many countries to validate this definition. In addition, we asked only about travel in the preceding year, and some infection control departments might only have asked about high-risk travel. We do not have molecular typing data for all isolates, which limits our ability to detect transmission within Canada. Similarly, we do not have data regarding the investigation of transmission or environmental reservoirs at individual hospitals. Although we have corrected for multiple comparisons, particular caution should be used in interpreting the statistical significance of comparisons with p values close to 0.05. We did not identify endoscopy as a risk factor for acquisition of CPE; however, our power to do so might have been limited, and exposure to outpatient endoscopy might not have been captured in patients with data from chart review only.

In conclusion, the incidence of CPE infection is increasing in south-central Ontario. Our data suggest that, even early on in the emergence of CPE, a substantial proportion of CPE infections are autochthonous cases, including most of those with KPC- and VIM-producing isolates. Policy and practice changes are needed to better protect patients from CPE exposure and acquisition in southern Ontario.

Technical Appendix Susceptibility testing of carbapenemase-producing *Enterobacteriaceae* from patients with clinical isolates, Metropolitan Toronto and the Regional Municipality of Peel, south-central Ontario, Canada, 2007–2015.
