# Supplementary material for: Emergence of Carbapenemase-Producing Enterobacteriaceae, South-Central Ontario, Canada
Source: Emerg Infect Dis. 2018 Sep;24(9):1674–82. doi: 10.3201/eid2409.180164 (PMC6106407; doi:10.3201/eid2409.180164)
Supplement: Technical Appendix — Susceptibility testing of carbapenemase-producing Enterobacteriaceae from patients with clinical isolates, Metropolitan Toronto and the Regional Municipality of Peel, south-central Ontario, Canada, 2007–2015. [file 18-0164-Techapp-s1.pdf]

Article DOI: https://doi.org/10.3201/eid2409.180164

Emergence of Carbapenemase-Producing *Enterobacteriaceae*, South-Central Ontario, Canada

Technical Appendix

Technical Appendix Table. Susceptibility testing of carbapenemase-producing *Enterobacteriaceae* from patients with clinical isolates (N = 149), Metropolitan Toronto and the Regional Municipality of Peel, south-central Ontario, Canada, 2007–2015

	No. (%)					
Drug	All patients, n = 149	NDM, n = 74	KPC, n = 43	OXA-48, n = 19	VIM, n = 13	p-value
Any carbapenem	8/135 (6)	2/69 (3)	2/35 (6)	4/18 (22)	0/11 (0)	**0.04**
Ertapenem	1/105 (1)	1/56 (2)	0/23 (0)	0/15 (0)	0/11 (0)	1.00
Meropenem	6/114 (5)	0/60 (0)	2/28 (7)	4/16 (25)	0/10 (0)	**0.002**
Imipenem	2/14 (14)	1/5 (20)	1/7 (14)	0/1 (0)	0/1 (0)	1.00
Any aminoglycoside	64/146 (44)	13/74 (18)	30/40 (75)	10/19 (53)	11/13 (85)	**<0.0001**
Gentamicin	43/142 (30)	8/72 (11)	24/38 (63)	3/19 (16)	8/13 (62)	**<0.0001**
Amikacin	23/99 (23)	6/64 (9)	6/18 (33)	7/11 (64)	4/6 (67)	**<0.0001**
Tobramycin	17/125 (14)	2/68 (3)	8/27 (30)	3/19 (16)	4/11 (36)	**0.0003**
Ciprofloxacin	18/131 (14)	2/69 (3)	9/38 (24)	3/14 (21)	4/10 (40)	**0.0003**
Cotrimoxazole	36/142 (25)	15/74 (20)	12/36 (33)	4/19 (21)	5/13 (39)	0.30
Nitrofurantoin	12/86 (14)	8/52 (60)	3/19 (16)	1/7 (14)	0/8 (0)	0.87
Colistin	15/17 (88)	9/10 (90)	3/4 (75)	3/3 (100)	–	0.67
Tigecyclin	15/29 (52)	9/20 (45)	3/5 (60)	3/4 (75)	–	0.63
Fosfomycin	8/10 (80)	7/9 (78)	1/1 (100)	–	–	1.00

*KPC, *Klebsiella pneumoniae* carbapenemase; NDM, New-Delhi metallo-β-lactamase; OXA-48, oxacillinase 48; VIM, Verona integron-encoded metallo-β-lactamase. Bold type indicates statistical significance (p<0.05).
